# Letter regarding 'Ethnicity and clinical outcomes in COVID-19: A systematic review and meta-analysis'

**DOI:** 10.1016/j.eclinm.2020.100685

**Published:** 2020-12-25

**Authors:** Richard Armitage

**Affiliations:** Division of Epidemiology & Public Health, University of Nottingham, Nottingham City Hospital, Hucknall Road, Nottingham NG5 1PB, UK

I read with interest the systematic review and meta-analysis by Sze et al. [1], which found that individuals of Black and Asian ethnicity are at increased risk of COVID-19 infection compared to White individuals.

Social distancing restrictions, community-wide lockdowns, and the wearing of personal protective equipment have been implemented globally in attempts to contain the spread of SARS-CoV-2, reflecting the knowledge that prolonged physical proximity and verbal interaction between individuals increases the likelihood of viral transmission [2]. Accordingly, key risk factors such as population density, occupational exposure, and household size must be taken into account when comparing the risk of COVID-19 infection between groups. Despite this, there was little consistency in the adjustments made for confounders in the studies included in this meta-analysis, with many making no adjustments whatsoever. The smaller effect sizes in the adjusted analyses compared to the unadjusted analyses highlight the importance of these confounding factors, and underscore the paramount importance of collecting, reporting, and including such data in statistical investigations of this nature. Clarity on this matter is urgently required as calls are made to consider ethnicity in the prioritisation of COVID-19 vaccine allocation [3], and structural racism is hypothesised to contribute to an increased risk of poor clinical outcomes in ethnic minority groups.^1^

## Declaration of Competing Interest

RA declares no competing interests.
